# PhageScanner: a reconfigurable machine learning framework for bacteriophage genomic and metagenomic feature annotation

**DOI:** 10.3389/fmicb.2024.1446097

**Published:** 2024-09-17

**Authors:** Dreycey Albin, Michelle Ramsahoye, Eitan Kochavi, Mirela Alistar

**Affiliations:** ^1^Department of Computer Science, University of Colorado at Boulder, Boulder, CO, United States; ^2^ATLAS Institute, University of Colorado at Boulder, Boulder, CO, United States

**Keywords:** bacteriophages, machine learning, phage virion proteins (PVP), protein prediction, deep learning, PVP identification

## Abstract

Bacteriophages are the most prolific organisms on Earth, yet many of their genomes and assemblies from metagenomic sources lack protein sequences with identified functions. While most bacteriophage proteins are structural proteins, categorized as Phage Virion Proteins (PVPs), a considerable number remain unclassified. Complicating matters further, traditional lab-based methods for PVP identification can be tedious. To expedite the process of identifying PVPs, machine-learning models are increasingly being employed. Existing tools have developed models for predicting PVPs from protein sequences as input. However, none of these efforts have built software allowing for both genomic and metagenomic data as input. In addition, there is currently no framework available for easily curating data and creating new types of machine learning models. In response, we introduce PhageScanner, an open-source platform that streamlines data collection for genomic and metagenomic datasets, model training and testing, and includes a prediction pipeline for annotating genomic and metagenomic data. PhageScanner also features a graphical user interface (GUI) for visualizing annotations on genomic and metagenomic data. We further introduce a BLAST-based classifier that outperforms ML-based models and an efficient Long Short-Term Memory (LSTM) classifier. We then showcase the capabilities of PhageScanner by predicting PVPs in six previously uncharacterized bacteriophage genomes. In addition, we create a new model that predicts phage-encoded toxins within bacteriophage genomes, thus displaying the utility of the framework.

## 1 Introduction

Bacteriophages (phages) are recognized as the most prolific organisms on Earth (Guerin and Hill, 2020; Brown et al., 2022). Given that phages play an integral role in shaping bacterial ecology (Braga et al., 2020; Dennehy and Abedon, 2021), they have emerged as a potential therapeutic against infections caused by antibiotic-resistant strains of bacteria. Phage therapy relies on phages to lyse and kill resistant bacteria rather than on antibiotics (Lekunberri et al., 2017). Some of the studies, case reports, and clinical trials that have tested phage therapy protocols (Liu et al., 2021) indicate possible issues such as endocarditis and non-lethal reversible transaminitis. Additionally, phages can also impact their bacterial hosts with effects such as (but not limited to) promotion of biofilm formation (*Pseudomonas aeruginosa* and phage pf4) or enhancing antibacterial resistance (*Salmonella spp*. and a P1-like phage) (Zajdowicz, 2022). That being said, there is a need for the development of quality control practices to popularize phage therapy as an alternative treatment for bacterial infections, and most importantly, ensure its safety and efficacy (Zajdowicz, 2022).

We focus this work on phage virion proteins (PVPs) and phage encoded toxins. PVPs are structural proteins (major capsid, tail fiber protein, etc.), and they contribute to phage-bacterial host interactions such as the infection process (i.e., a tail fiber protein is responsible for a phage's recognition and attachment to a viable bacterial host) (Boeckaerts et al., 2021). Phage encoded toxins are virulence factors that are encoded into the bacterial host's genome when the phage integrates its own DNA. While being lysed the bacterial host can release these toxins, allowing them to further damage the host organism (a human or animal) (Abedon and Lejeune, 2007).

As phage therapy involves both the process of recognition and infection, as well as possible outcomes from the lysing process itself, this motivates interest in identifying both PVPs and phage encoded toxins to further improve efficacy and safety of phage therapy (Abedon and Lejeune, 2007; Fang et al., 2022).

Identifying PVPs traditionally involves experimental strategies comprising of bioprotocols such as mass spectrometry (Lavigne et al., 2009) and protein arrays (Jara-Acevedo et al., 2018; Yuan and Gao, 2016; Mwale et al., 2020); consequently, these techniques are labor-intensive and time-consuming (Kabir et al., 2022; Meng et al., 2020). Therefore, there is a growing interest in leveraging *computational techniques* to expedite the identification of PVPs (Kabir et al., 2022; Meng et al., 2020).

Computational support in PVP identification first gained traction in 2012 when Seguritan et al. utilized feed-forward neural networks to predict proteins as either structural or non-structural class (i.e., binary classification) (Seguritan et al., 2012). To determine the accuracy of this computational approach, the researchers examined the input proteins using transmission electron microscopy (TEM). The tested protein set was comprehensive and includes both capsid proteins and tail fiber proteins. The most accurate prediction (over 80%) was obtained by a model comprising of 160 networks that classified proteins through a majority voting scheme (Seguritan et al., 2012).

Encouraged by the success of Seguritan's approach, researchers focused next on improving the binary classification of phage proteins either as PVPs or as not PVPs, and used machine learning (ML) algorithms like random forests (RFs) (Ahmad et al., 2022), support vector machines (SVMs) (Manavalan et al., 2018), naive Bayes (NB) (Feng et al., 2013), or ensemble methods (Barman et al., 2023). In 2020, Cantu et al. (2020) released “PhANNs,” a software that used multiclass classification to identify the specific type of PVP among 10 different classes. Two years later, DeePVP, proposed by Fang et al. (2022) built upon this approach by employing a convolutional neural network (CNN) for PVP type identification. Leveraging the PhANNs' dataset, this work enhanced prediction performance for both binary and multiclass PVP prediction. These approaches were limited to directly using protein sequences, ignoring the potential of genomic or metagenomic data.

**Figure 1:**
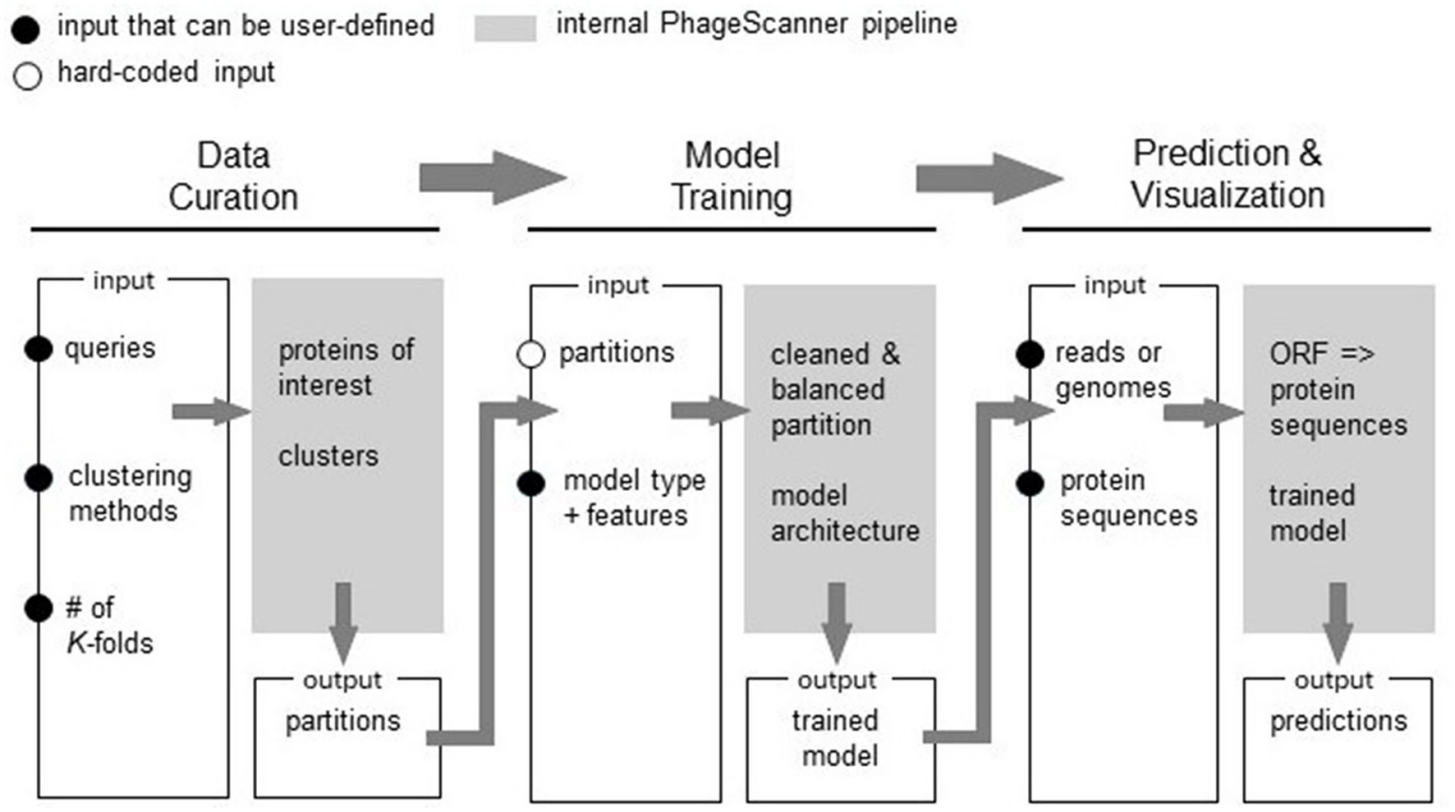
Overview of the PhageScanner Machine Learning (ML) Pipeline. The ML pipeline is composed of three interconnected phases: (1) Data Curation, (2) Model Training, and (3) Prediction and Visualization. Each step is designed to be executed from the command line, and each features a customizable configuration file. The process of model curation is split into two stages. The Data Curation phase is responsible for curating data through adapters connected to a protein database. Subsequently, Model Training phase extracts features and initiates model training as defined by the corresponding configuration file. The Prediction and Visualization phase employs the trained model to predict bacteriophage protein classes found in genomes, metagenomes, or proteins.

Our research extends upon these studies by introducing PhageScanner, an open-source tool that empowers users to easily create classifiers from various input sources. As schematically shown in Figure 1, PhageScanner is designed to be modular and easily reconfigured by the user at the data curation, model creation and training, and the prediction levels. Unlike existing software which may only have one classification mode or limited accepted input types, PhageScanner offers both binary and multiclass detection capabilities, as well as accepting full genomes, protein sequences, and metagenomic sequencing inputs. In this paper, we demonstrate PhageScanner for the purpose of PVP and phage-encoded toxin prediction.

First, the user can configure the data such that it is retrieved from Uniprot and/or Entrez databases. Next, PhageScanner can be run with user-defined models and feature extraction methods. At the prediction level, PhageScanner integrates an existing workflow for annotating Open Reading Frames (ORFs) within genomic and metagenomic data. The code is released open-source, allowing the community to contribute to the existing source code for new machine learning models and methods. This includes adjusting any part of the pipeline to implement a different approach. Our user-focused approach extends beyond the expert user (i.e., that may be able to edit the code). Specifically, at the input level, we provide the user with configuration files that can be used to reproduce the models presented here, or to create new models based on protein classes of interest (e.g., “Toxic Proteins”). At the output level, PhageScanner is paired with a graphical user interface (GUI) that enables the user to visually scrap proteins of interest.

Apart from the user-centric approach, PhageScanner demonstrates algorithmic improvements, as follows. We expanded PhageScanner such that it can curate data from both UniProt and Entrez. Due to the complexity of data curation in general, past work has been focused on either one of the two databases, e.g., only utilize Uniprot to source ground truth proteins (Manavalan et al., 2018; Feng et al., 2013; Ahmad et al., 2022), or rely exclusively on proteins obtained from Entrez (Cantu et al., 2020; Fang et al., 2022). To enable a direct comparison between existing approaches, which is essential for community engagement and further community-based development of the open-source tool, PhageScanner incorporates a BLAST classifier. This allows for a direct comparison between previous ML-based classifiers and solutions using sequence alignment.

As such, we use PhageScanner for testing models for prediction of both binary and multiclass PVPs. We also use PhageScanner as a tool to predict phage-encoded toxins, which may help in ensuring the safety of future phage therapies. Last, we propose PhageScanner as a simplified framework for data collection, model training, model testing, and PVP prediction; this is shown with PhageScanner's ability to explore genomic and metagenomic data and with its easy-to-use GUI.

The source code for the pipelines and the GUI, as well as pre-trained models, are available at https://github.com/Dreycey/PhageScanner.

## 2 Materials and methods

When running in its default mode, PhageScanner curates a dataset based on user choices and interests, and it creates and trains a machine learning models based on the user selection of 9 available ML models. Moreover, we implemented PhageScanner to easily integrate a user's preexisting model, and to utilize the trained model based on the user's reads, genomes, or protein sequences. PhageScanner outputs classification predictions as a CSV file and within an interactive GUI.

The wide variety of PhageScanner's functionality is a result of both our own contributions and existing popular bioinformatics and machine learning frameworks such as CD-HIT (Fu et al., 2012), Megahit (Li et al., 2015), BLAST (Altschul et al., 1997), PHANOTATE (McNair et al., 2019), Keras (Gulli and Pal, 2017), and Scikit-Learn (Pedregosa et al., 2011). Our contributions include easy customization through configuration files, error-handling, and logging to provide users with insight into code progress. We also created custom classes to allow for seamless integration of a user's own ML model architecture using a custom configuration file.

We distinguish between three big phases of the process: data curation, model training and PVP prediction. We decouple data curation from model training to enable a modular approach that can reduce the runtime needed to iterate through user-defined models. With PhageScanner, data curation has to be run once, while model training and testing can be run multiple times to compare results from various models or to wrap the prediction step into a seperate pipeline. The last phase, protein prediction, is complemented by a visual GUI that allows post-processing of the results even by external people (supervisors, collaborators).

### 2.1 Phase 1: data curation

PhageScanner can take multiple protein databases as input [specifically only Uniprot (Consortium, 2015), only Entrez (Maglott et al., 2005), or both] as opposed to being limited to just one database like previously mentioned tools. It is important to note that Uniprot has the advantage of containing experimentally validated proteins, while Entrez may contain unvalidated proteins. In our experiments, we utilize both databases. We specified the Entrez queries to ignore proteins categorized as “hypothetical,” “probable,” “unknown,” or “putative.” This was an approach used by PhANNs (Cantu et al., 2020), and we incorporated this usage into our database pipeline to ensure all proteins retrieved automatically ignore non-reliable proteins.Model performance, especially for larger models such as the LSTM-RNN, is dependent on the amount of training data available. We leave the choice of what databases to use to the user's discretion.

First, the proteins are retrieved from the input databases based on the targeted prediction class. When we tested PhageScanner on binary PVP prediction, the queries gathered proteins based on various criteria as shown in the configuration files specifications shown below:


classes :
  - name: PVP
   uniprot: ‘‘capsid AND
   cc_subcellular_location: virion
   AND reviewed: true''
  - name: non-PVP
   uniprot: ‘‘capsid NOT
   cc_subcellular_location: virion
   AND reviewed: true''

After retrieval, proteins are clustered with the Cluster Database at High Identity with Tolerance (CD-HIT) program (Fu et al., 2012) using an identity threshold defined in the corresponding configuration file, as shown below:


clustering :
  deduplication-threshold: 100
  clustering-percentage: 90
  k_partitions: 5

Following this, each protein cluster is divided into *K* partitions for performing *K*-fold cross-validation. One partition is allocated for testing, and the remainder for training. We used *K* = 5 (value can be user-adjusted) to align with prior work (Rodriguez et al., 2009).

### 2.2 Phase 2: model training

To complete model training and testing, the partitions and user specifications are obtained as input. We removed all non-canonical amino acids, as defined by Young and Schultz (2010), and processed the protein sequences by removing all non-canonical amino acids from the protein sequences. The features specified in the configuration file are then extracted from each protein and these extracted features are used to train downstream models using the *K*-fold cross-validation partitions created during data curation. Our method for machine-learning-based PVP identification aligns with prior research (Meng et al., 2020).

Prior work demonstrating the use of machine learning models for predicting PVPs used several different methods for obtaining feature vectors from proteins (Seguritan et al., 2012; Ahmad et al., 2022; Manavalan et al., 2018; Feng et al., 2013; Barman et al., 2023). These feature vectors encode the sequence and functional information of the protein in a format required for machine learning models. We explain how we decided which feature extraction method to use in Section 3.1.

In PhageScanner, we employ a factory design pattern (Welicki et al., 2008), which is a software design approach that facilitates the combination of different extracted features **f_*n*_(*p*_*i*_)** into a more comprehensive feature vector **F(*p*_*i*_)** (Equation 1). This allows for the user to easily create their own combinations out of the available features. All available features are further described at https://github.com/Dreycey/PhageScanner.


(1) $$F(p_{i})=\left[\begin{array}{c} f_{1}(p_{i})\\ f_{2}(p_{i})\\ \vdots \\ f_{n}(p_{i}) \end{array}\right]$$

Next, the user can select a model from multiple options: they can embed their own model architecture or they can modify skeletons provided by PhageScanner. If the user chooses to enter their own model architecture, they can utilize the base PhageScanner, Sci-kit Learn, or Keras Model class templates which include several intuitive class functions (load, predict, test, save, etc.). Alternatively, PhageScanner provides the following model skeletons in Table 1 that can be modified by the user.

**Table 1:** Overview of models available in PhageScanner.

**Name**	**Configuration file specifier**
Support vector classifier	SVC
Multinomial naive bayes classifier	MULTINAIVEBAYES
Logistic regression classifier	LOGREG
Gradient boosting classifier	GRADBOOST
Random forest classifier	RANDOMFOREST
Feed-forward neural network classifier	FFNN
Recurrent neural network multiclass classifier	RNN
Convolutional neural network	CNN
BLAST classifier	BLAST

All of the model architectures available in the PhageScanner package. Each model comes with a unique specifier that can be referenced within the input configuration file used for the tool.

Finally, using the partitions derived from the data curation step, the selected feature vector, and the user's chosen model, PhageScanner trains and tests the selected model. PhageScanner also provides functionality to save model results after *K*-fold cross validation into a CSV file (time execution, confusion matrix, F1-score, precision, recall, accuracy, etc.) and also saves the trained model.

### 2.3 Phase 3: prediction and visualization

PhageScanner can utilize the user's desired trained model to make predictions about protein sequences. It can accept input as either reads, genomes, or protein sequences. The user's desired trained model is then used for prediction, with the final output being a CSV file containing accession IDs, lengths, start and stop positions, ORF scores, associated protein sequences, and the predicted classes.

**Figure 2:**
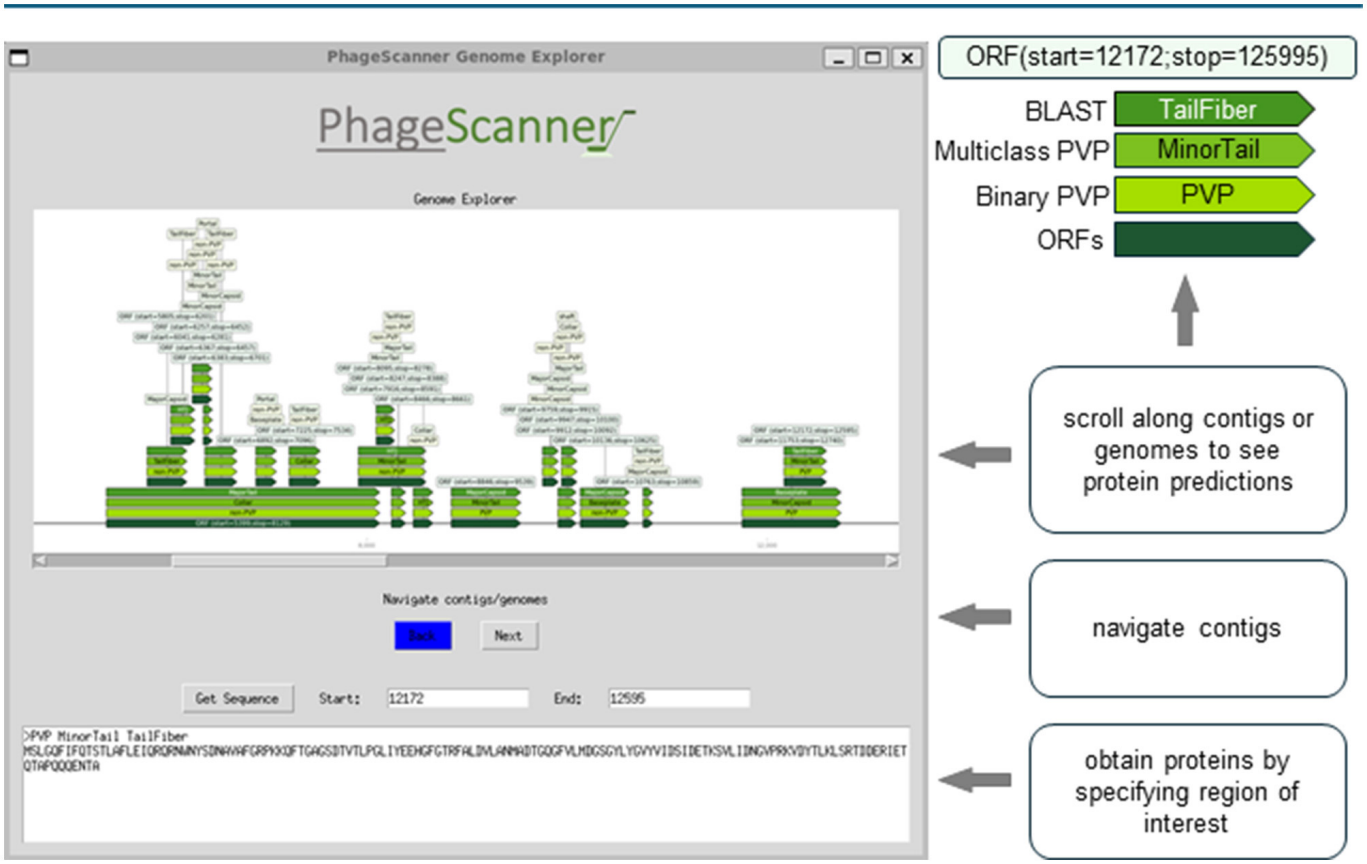
Graphical user interface (GUI) for visual assessment of genomic and metagenomic annotations. The Prediction phase produces a comma-separated (CSV) summary of features found from genomic and metagenomic annotations. This output can be studied using an open-source, graphical user interface (GUI). The GUI provides a method for end-users to easily navigate across different annotations while cross-referencing models with BLAST. The contigs and/or genomes can be scrolled through, and the retrieval of proteins specified within a genomic range can be analyzed. The example depicted can be run from the sample run instructions available at https://github.com/Dreycey/PhageScanner.

For visualization, PhageScanner uses a combination of Python based libraries, such as TKinter (Lundh, 1999) and the DNA Features Viewer library (Zulkower and Rosser, 2020). As shown in Figure 2, each used model (BLAST, Multiclass PVP, and Binary PVP) is displayed on a separate row and in a different color along the contig or genome and the ORFs created from PHANOTATE are displayed at the bottom. The user can explore visually the annotated contigs or genomes from the output CSV file.

### 2.4 Feature extraction method selection and performance

For feature extraction, PhageScanner uses techniques commonly applied for PVP identification (Meng et al., 2020). These include the extraction of dipeptide (“DPC”; 200 features) and tripeptide (“TPC”; 8000 features) frequencies from protein inputs. It also includes Biopython-based (Cock et al., 2009) methods to derive chemical features and isoelectric points (“ISO”). Expanding on the “PVP” vector used by PVP-SVM (Manavalan et al., 2018), the 16-element “CHEMFEATURES” vector includes information on polarity, hydrophobicity, aromaticity, charge information, protein size, mass, isoelectric point, and secondary structure frequencies. The atomic composition vector (“ATC”) contains five elements representing frequencies of Hydrogen, Carbon, Nitrogen, Oxygen and Sulfur in the protein. For CTD and PseudoAAC, we utilize PyBioMed for the implementation (Dong et al., 2018). The “AAC” measures the frequency of each amino acid type in the protein, resulting in a 20-element feature vector.

We evaluated each feature extraction method for accuracy and timing using a baseline one-vs-all logistic regression classifier, as outlined in Table 2. In addition to the performance metrics (F1 Score, Precision, Recall), the execution time is displayed to compare how the vector sizes impact model inference timing. Execution time is of particular importance when using PhageScanner on large metagenomic datasets.

**Table 2:** Performance metrics for various feature combinations.

**Feature set**	**F1 score**	**Precision**	**Recall**	**Execution time (s)**
AAC	0.725	0.865	0.732	0.000728
ATC	0.384	0.668	0.600	0.000875
ATC + CTD + AAC + ISO + CHEMFEATURES	0.790	0.892	0.721	0.002515
CHEMFEATURES	0.572	0.899	0.600	0.000973
CHEMFEATURES + DPC (gap_size=0)	0.574	0.902	0.640	0.004715
CTD	0.818	0.885	0.765	0.002101
DPC (gap_size=0)	0.823	0.895	0.865	0.005625
DPC (gap_size=0) + ATC + CTD	0.811	0.895	0.760	0.008700
DPC (gap_size=0) + TPC	0.873	0.905	0.942	0.112359
DPC (gap_size=1)	0.835	0.885	0.832	0.004104
DPC (gap_size=9)	0.818	0.895	0.848	0.004733
ISO	0.386	0.882	0.286	0.000815
TPC	0.909	0.912	0.981	0.063712

Presented are the metrics for the feature combinations used in model analysis, including the F1 score, precision, recall, and execution time in seconds for each feature set.

**Figure 3:**
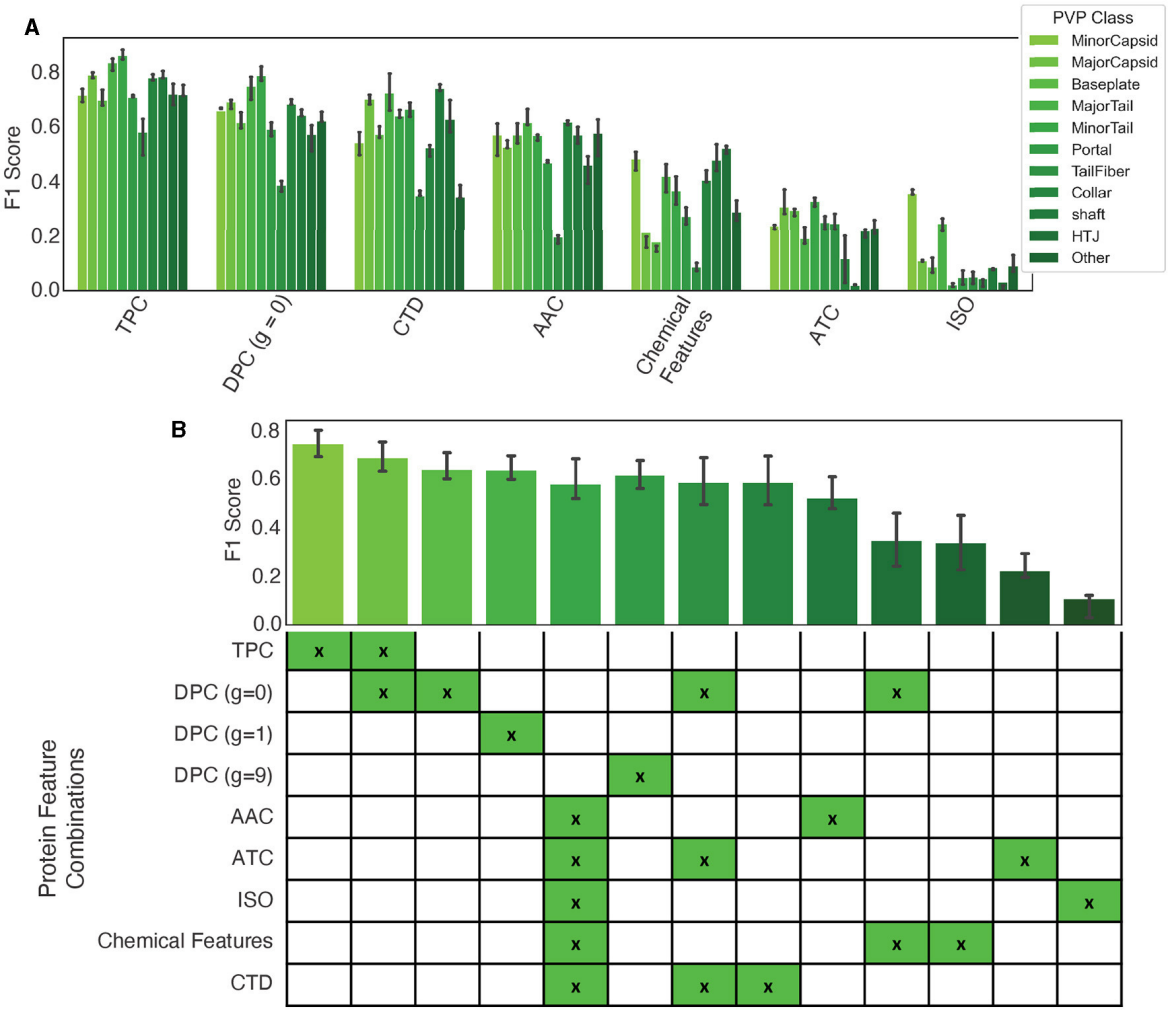
Protein feature extraction testing. **(A)** The F1 scores for standalone features using a baseline logistic regression (one-vs-all) classifier. Colors correspond to the specified PVP class. **(B)** Mean F1 scores among all classes for different feature combination approaches.

Our findings are presented in Figure 3A, and show that dipeptide and tripeptide frequency features were the most accurate for multiclass PVP prediction with a F1 score of 87%. Additionally, tail fiber proteins were the most difficult to predict with noticeably lower F1 scores for the model utilizing the following standalone features: TPC, DPC (*g* = 0), CTD, AAC, and Chemical Features. When we combined features into a concatenated vector, as shown in Equation 1, the combination of dipeptide and tripeptide frequencies resulted in the highest F1 score out of the combinatorial-predictors (87% shown in the mean F1 scores among all classes in Figure 3B). Baseline models using feature combinations had higher F1 scores compared to models using one feature. However, the combination of dipeptide and tripeptide features required the more time for extraction, due to the large feature vector size (8,400 elements)—a crucial consideration for users wishing to use PhageScanner for extensive metagenomic dataset analysis.

## 3 Results

To demonstrate the capabilities of PhageScanner, we utilize each phase (data curation, training, and prediction) to perform PVP identification via both multiclass and binary models. We discuss varying performance when comparing different features. We perform benchmark testing comparing PhageScanner models to existing PVP identification models. We also create a new model for predicting phage-encoded toxins.

### 3.1 System requirements and model architectures

PhageScanner is primarily available for Linux and Mac, but provides support for Windows via Docker and WSL. The model architectures for the Multiclass Support Vector Classifier (SVC), Multinomial Naive Bayes Classifier, Logistic Regression Classifier, Gradient Boosting Classifier, and Random Forest (RF) Classifier used in the experiments are outlined in Table 3. The architectures for the Feed-forward Neural Network (FFNN) Classifier, Long Short-Term Memory (LSTM) Recurrent Neural Network (RNN) Multiclass Classifier, and Convolutional Neural Network (CNN) [based on DeePVP (Fang et al., 2022)] are listed below, respectively. For more architecture details about each model as well as the PhageScanner's in-house BLAST classifier, refer to the “models.py” file in the main folder at https://github.com/Dreycey/PhageScanner.

**Table 3:** Model details and altered parameters.

**Model name**	**Model details**	**Altered parameters**
Multiclass support vector classifier (SVC)	Default	random_state = 0 tol = 1e-5 probability = True
Multinomial naive bayes classifier	Default	force_alpha = True
Logistic regression classifier	Default	random_state = 0 multi_class = “ovr”
Gradient boosting classifier	Default	random_state = 0 n_estimators = 10 learning_rate = 0.1
Random forest classifier	Default	random_state = 0 max_depth = 10

Each model used for binary classification, including the specific model details and the parameters that were altered.

#### 3.1.1 Feed-forward neural network architecture

1. **Input layer**

- Description: Dense layer with ReLU activation
- Parameters:

° Units: 100
° Activation: ReLU
° Input Shape: feature_vector_length
° Kernel Initializer: random_uniform

2. **Hidden layer 1,3,5**

- Description: Dropout layer
- Parameters:

° Rate: 0.2

3. **Hidden layer 2,4**

- Description: Dense layer with ReLU activation
- Parameters:

° Units: 200
° Activation: ReLU

4. **Output layer**

- Description: Dense layer with softmax activation
- Parameters:

° Units: number_of_classes
° Activation: Softmax

5. **Compilation**

- Optimizer: Adam

° Learning Rate: 0.001
° Beta 1: 0.9
° Beta 2: 0.999
° Decay: 0.0
° AMSGrad: False

6. **Loss function**

- Sparse Categorical Crossentropy

7. **Metrics**

- Accuracy

### 3.2 Recurrent neural network multiclass classifier architecture

1. **Input layer**

- Description: LSTM layer
- Parameters:

° Units: 50
° Input Shape: (row_length, column_length)
° Return Sequences: False

2. **Hidden layer 1**

- Description: Dense layer with ReLU activation
- Parameters:

° Units: 1000
° Activation: ReLU

3. **Hidden layer 2**

- Description: Dense layer with ReLU activation
- Parameters:

° Units: 100
° Activation: ReLU

4. **Output layer**

- Description: Dense layer with softmax activation
- Parameters:

° Units: number_of_classes
° Activation: Softmax

5. **Compilation**

- Optimizer: Adam

° Learning Rate: default
° Beta 1: default
° Beta 2: default
° Decay: default
° AMSGrad: default

6. **Loss function**

- Sparse Categorical Crossentropy

7. **Metrics**

- Accuracy

### 3.3 Convolutional neural network model architecture

1. **Convolutional layer**

- Description: Conv1D layer with ReLU activation
- Parameters:

° Filters: 32
° Kernel Size: 3
° Activation: ReLU
° Input Shape: (row_length, column_length)

2. **Max pooling layer**

- Description: GlobalMaxPooling1D layer
- Parameters:

° Pool Size: 4

3. **Batch normalization layer**

- Description: BatchNormalization layer

4. **Hidden layer**

- Description: Dropout layer
- Parameters:

° Rate: 0.55

5. **Flatten layer**

- Description: Flatten the output from the previous layer

6. **Fully connected layer**

- Description: Dense layer with ReLU activation
- Parameters:

° Units: 64
° Activation: ReLU
° Kernel Regularizer: l1(0.01)

7. **Output layer**

- Description: Dense layer with softmax activation
- Parameters:

° Units: number_of_classes
° Activation: Softmax

8. **Compilation**

- Optimizer: Adam

° Learning Rate: default
° Beta 1: default
° Beta 2: default
° Decay: default
° AMSGrad: default

9. **Loss function**

- Sparse Categorical Crossentropy

10. **Metrics**

- Accuracy

**Figure 4:**
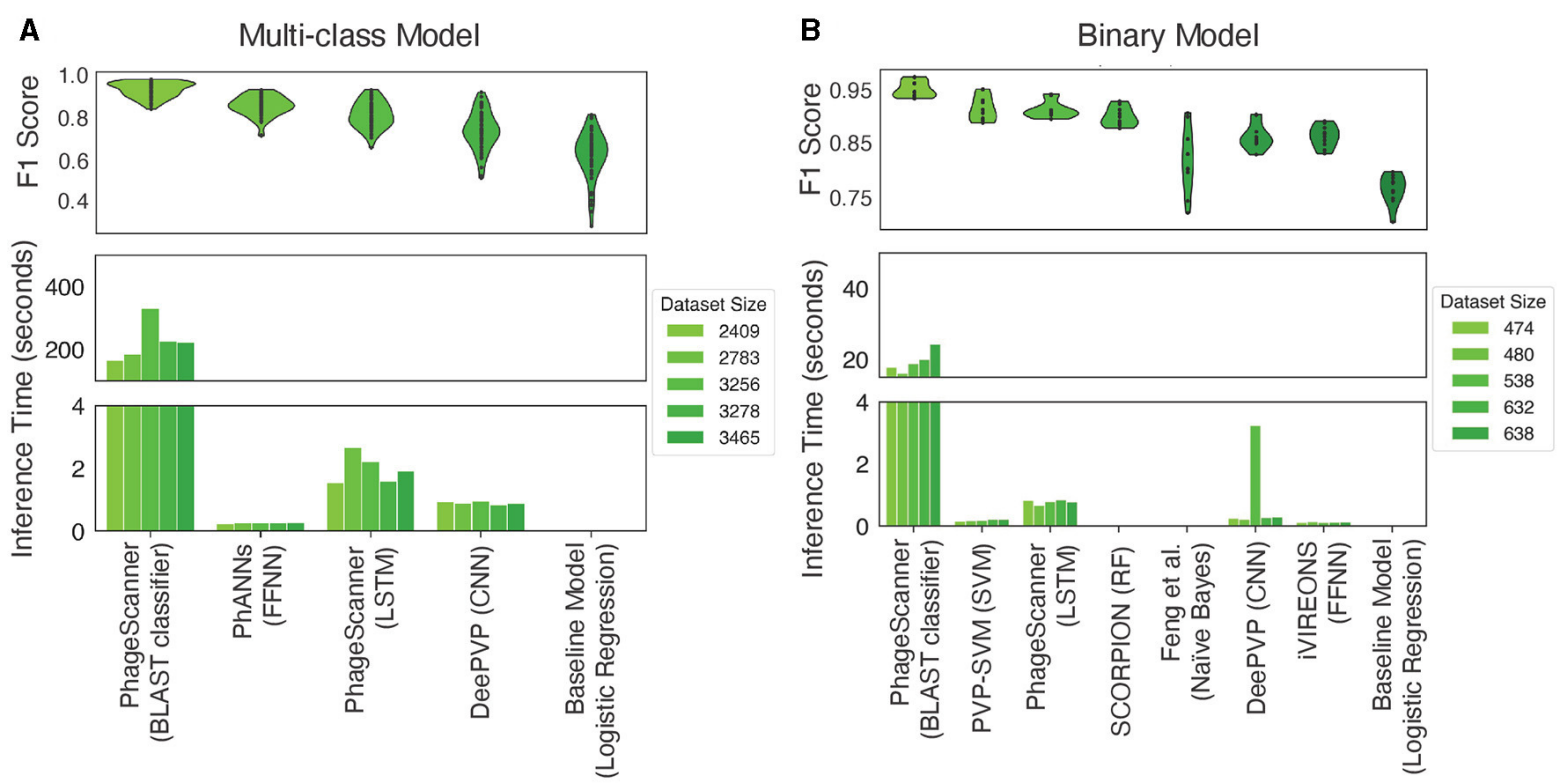
Testing performance among models for different classification tasks. **(A)** In the context of multiclass PVP prediction, the upper panel displays the F1 score across various models, while the lower panel illustrates the inference time of different multiclass classifiers. Inference timing was performed on dataset sizes, post clustering, of 2,409, 2,783, 3,256, and 3,465 proteins. **(B)** For binary PVP prediction, the upper panel demonstrates the F1 score across different models, whereas the lower panel showcases the inference among various binary classifiers. Inference timing was performed on dataset sizes, post clustering, of 474, 480, 536, 632, and 838 proteins.

Machine learning and deep learning models are shown to have the capability of providing additional information to existing methods of protein annotation (Seguritan et al., 2012; Ahmad et al., 2022; Manavalan et al., 2018; Hochreiter and Schmidhuber, 1997; Lecun et al., 1998; Bileschi et al., 2022), and PhageScanner seeks to provide users a streamlined framework for users to easily approach building their own machine learning and deep learning models. Thus, we implemented PhageScanner to enable both building-from-scratch as well as integration of existing architectures that can be adjusted specifically to the PVP identification and phage-encoded toxin identification (the results for 9 different architectures are presented in Figure 4). Generally, a model's success is dependent on various factors such as the task given, the data provided, the model's complexity, hyperparameter tuning, and so on. Our experimental setup primarily built upon the LSTM-RNN (Hochreiter and Schmidhuber, 1997), CNN (Lecun et al., 1998), and BLAST (Altschul et al., 1990) classifier models as they showed the highest performance in our preliminary tests (a comparison is shown in Figure 4). Our insights as to why these specific models may outperform the others, are based on the following observations:

- LSTM-RNNs are generally well suited for sequential data, handling sequences of various lengths, and maintaining context over long sequences.
- CNNs can learn spatial hierarchies of features via backpropagation as they were originally designed for use in images and videos.
- The BLAST classifier utilizes the BLAST algorithm, widely used to find regions of similarity between a provided input protein or nucleotide sequence and a database of known sequences.

Generally, a BLAST classifier is considered the first choice for most bioinformatics research, while both LSTM-RNNs and CNNs require additional data pre-processing, user customization, and training to perform well. However, we see the value in integrating LSTM-RNNs and CNNs as they can learn more complex patterns and thus provide more flexibility to researchers.

### 3.4 Experiments

To demonstrate the capabilities of PhageScanner, we perform the following experiments previously used for PVP prediction: (1) multiclass prediction (for 10 PVP proteins as per Cantu et al., 2020), and (2) binary prediction (determining if a protein is PVP or not as per Seguritan et al., 2012). We also (3) create a new model for binary prediction of toxic PVPs within phage genomes.

#### 3.4.1 Multiclass PVP prediction

**Figure 5:**
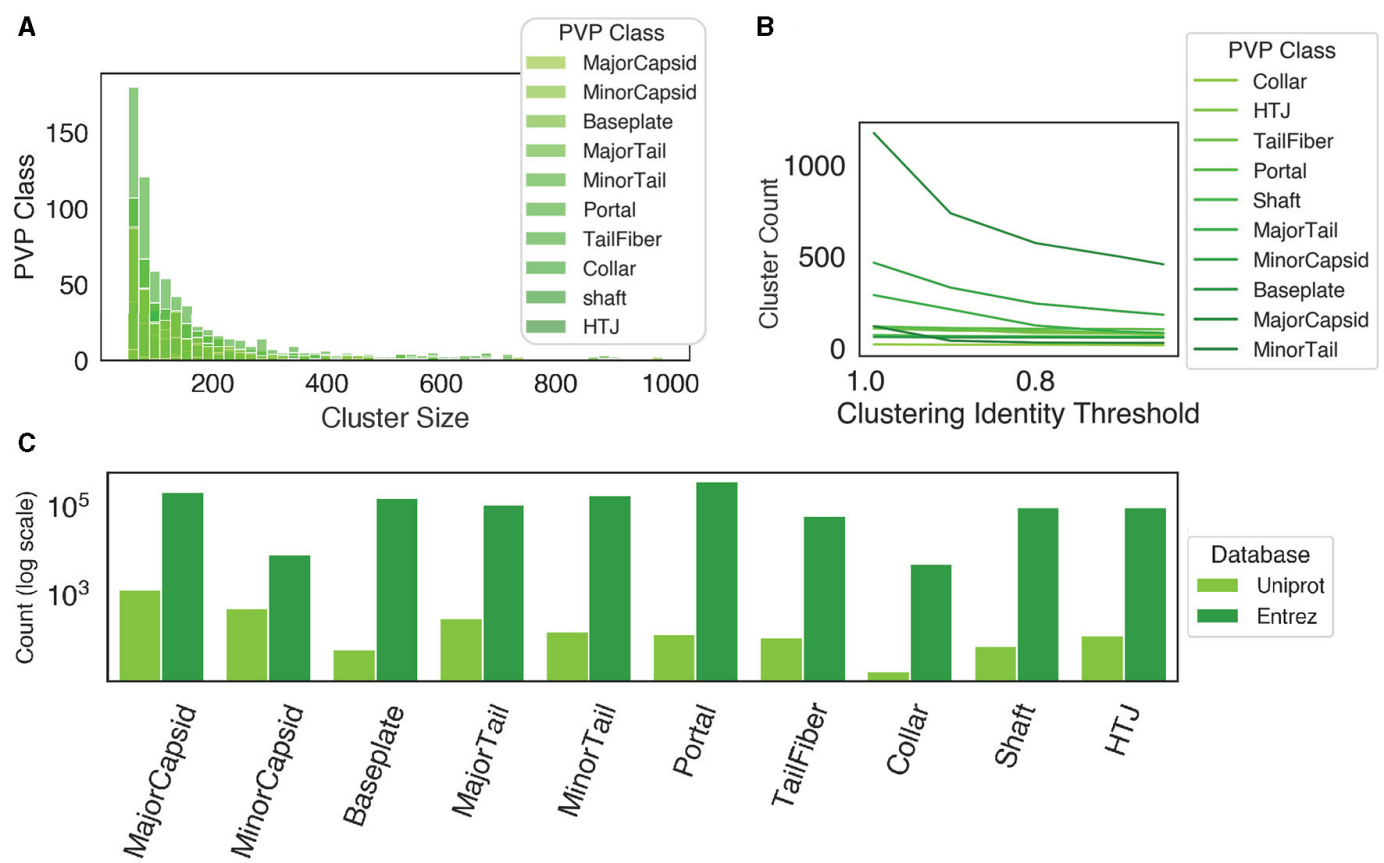
Database retrieval and clustering. **(A)** When the proteins are clustered at an identity threshold of 90% using CD-HIT, all protein classes have a higher frequency of small cluster sizes, while few clusters are larger than 400 proteins. **(B)** The number of clusters at different identity thresholds using CD-HIT. **(C)** The count of proteins from both Uniprot and Entrez before clustering.

When we applied PhageScanner's data curation step to collect proteins corresponding to the 10 PVP classes (Fang et al., 2022; Cantu et al., 2020), we find the following key observations. For clarification, the 10 classes contain 9 known PVP classes, and 1 “Other” class, denoting a non-PVP. Initially, at a clustering threshold of 90%, most clusters are composed of fewer than 400 proteins (Figure 5A). When the identity threshold for clustering is decreased more strict (i.e., decreased), we observe a declining trend in the number of total clusters (Figure 5B); this trend is most notable when the identity drops from 100% to 90%. These findings suggest more proteins belonging to smaller-sized clusters and it indicates the existence of unique proteins within these queries. In addition, there is a substantial difference in the initial count of proteins between Uniprot and Entrez; Entrez holds a significantly higher count of proteins per class compared to Uniprot (Figure 5C). As such, using a combination of Uniprot and Entrez along with clustering (as PhageScanner does) offers an advantage in creating larger datasets for downstream model training.

We then compared the performance of the BLAST classifier and LSTM against a reimplementation of PhANNs' feed-forward neural network (FFNN) and DeePVP's convolutional neural network (CNN). Each model was incorporated as an option in PhageScanner by either referencing model descriptions in the corresponding manuscripts or source code (when available, see Table 6). To do so, we employed a logistic regression one-vs-all classifier as a baseline. Upon evaluation, the BLAST classifier surpassed other PVP prediction methods in accuracy (94%), with the FFNN (86%) and LSTM (82%) models following behind (Figure 4A-top). However, the BLAST classifier's inference time was significantly higher than the other prediction models (Figure 4A-bottom).

##### 3.4.1.1 Testing PhageScanner on characterized bacteriophage genomes

**Figure 6:**
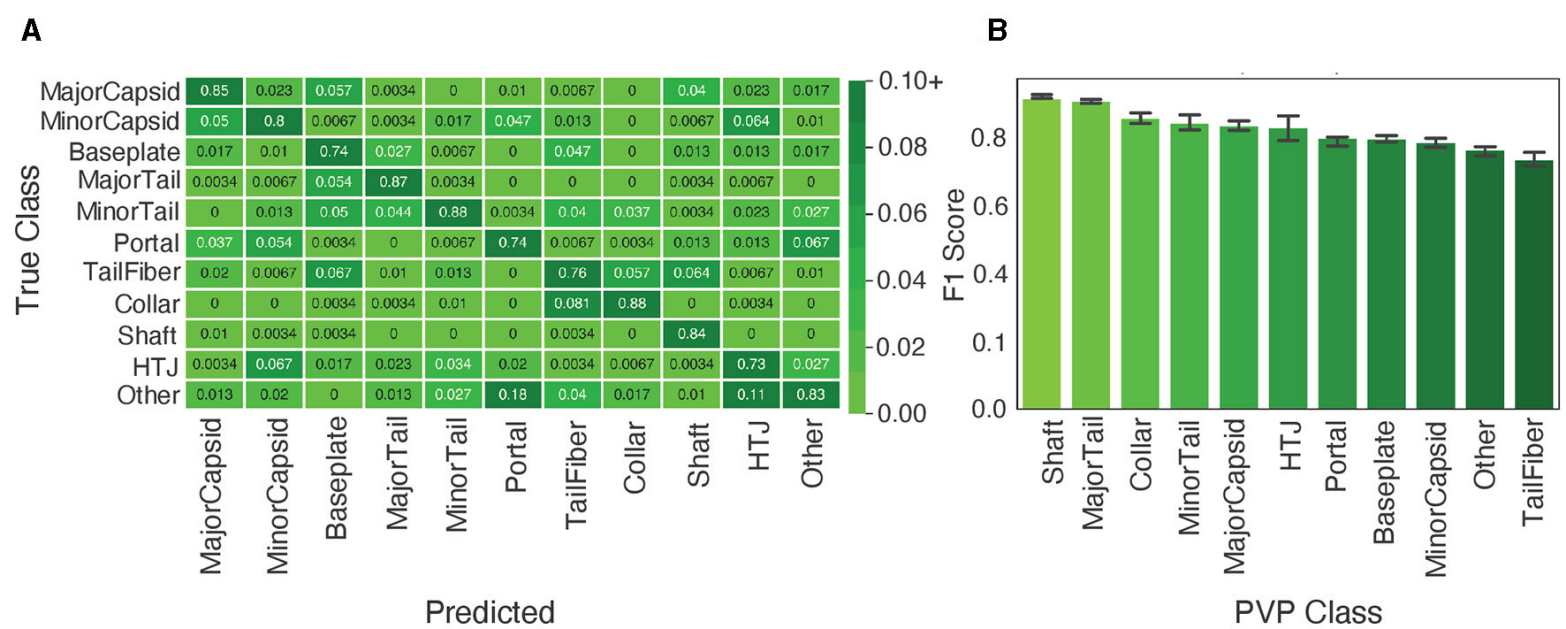
Analysis of the multiclass performance of PhageScanner on PVPs. **(A)** A confusion matrix displaying the frequency of the ground truth class (rows) to the predicted class (columns). The cell color spectrum is adjusted to range between 0 and 0.1. **(B)** The F1 score per class for the LSTM model, with the highest scoring classes arranged in descending order of F1 score.

As outlined, PhageScanner incorporates a BLAST classifier and an LSTM model for predicting PVP classes. However, it struggles to accurately classify certain PVP classes, as illustrated by the performance gap between it and the BLAST classifier (91% vs. 94%). Further exploration into the LSTM's performance per PVP class revealed a few challenging categorizations (Figure 6A), specifically with the model misclassifying Portal proteins as Minor Capsid proteins, and Tail Fiber proteins as Collar proteins (and vice versa). Such misclassifications are expected though, due to similar functions of the proteins. These misclassifications are also seen in the work of PhANNs (Cantu et al., 2020).

Furthermore, a significant number of proteins are falsely identified as the negative class “Other.” This issue may be rooted in PVPs being contained within the protein dataset corresponding to the “Other” class. It can potentially be addressed by refining the database queries. Figure 6A showcases the normalized confusion matrix for the LSTM classifier for multiclass PVP prediction. Interestingly, for the “Other” class, the model mistakenly predicts “Portal” and “HTJ” classes the most often (18% and 11%, respectively). Our analysis indicates the LSTM struggles most with the “Other” category and “Tail Fibers” class. This conclusion is based on the comparison of aggregated F1 scores across each PVP class (Figure 6B).

##### 3.4.1.2 Testing PhageScanner on the characterized *Mycobacteriophage* PDRPxv Genome

PhageScanner was used to annotate the coding sequence (CDS) regions in the *Mycobacteriophage* PDRPvx Genome to evaluate end-to-end performance. We chose the *Mycobacteriophage* PDRPvx Genome, a well-characterized reference genome commonly used in PVP prediction analysis (Cantu et al., 2020; Fang et al., 2022), because of its experimentally inferred putative functions performed by Sinha et al. (2020). Using the assembled genome (GenBank accession KR029087) directly, we were able to compare the GenBank annotations with the predictions from the PhageScanner LSTM-RNN, PhANNs, and BLAST Classifier models.

**Figure 7:**
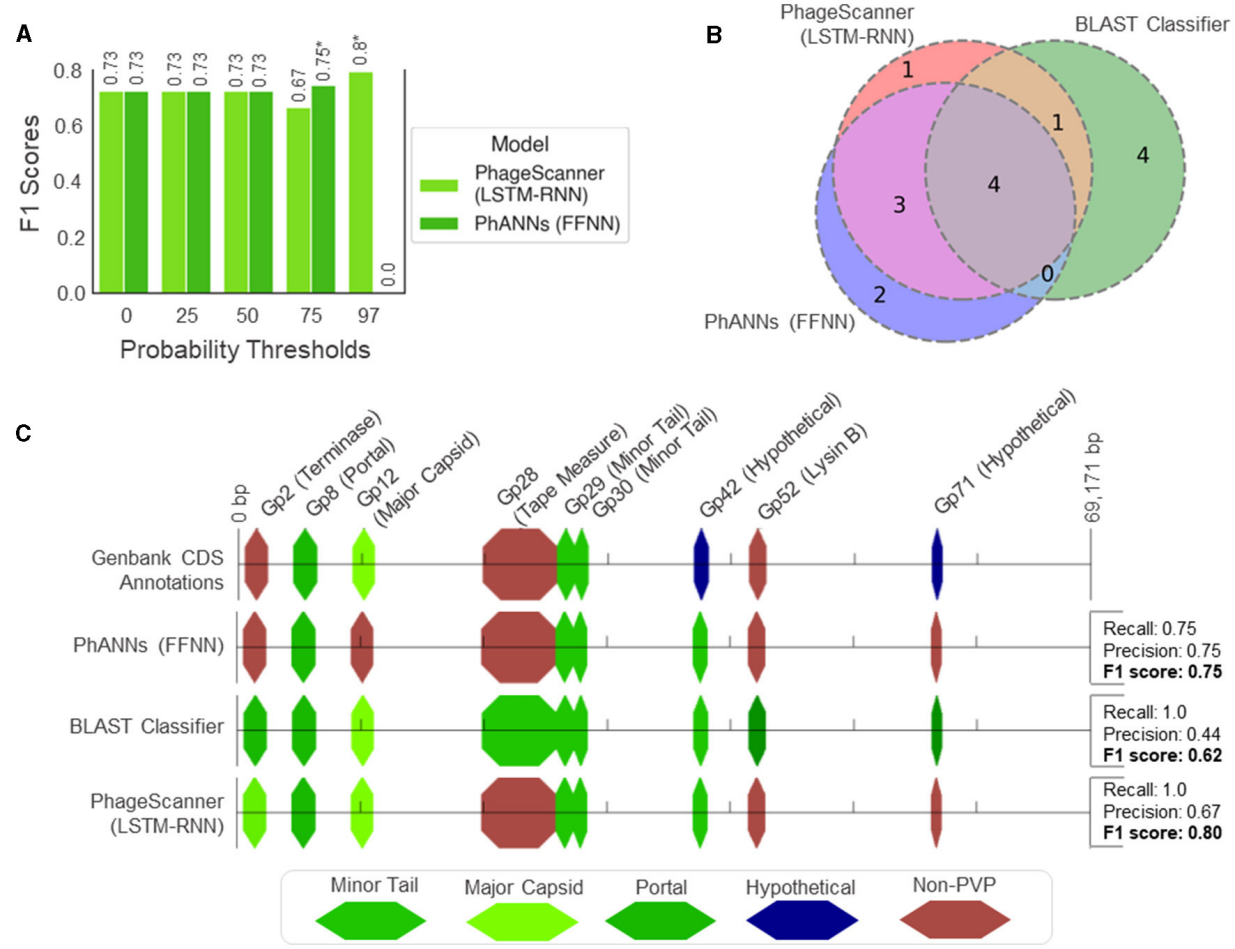
Genomic analysis of PVPs in the characterized *Mycobacteriophage* PDRPxv Genome. **(A)** Evaluation of model performance on the *Mycobacteriophage* PDRPxv genome at different probability thresholds for a positive PVP classification (otherwise, classified as “Non-PVP”). The F1 scores are shown above each bar and an asterisk (*) denotes the top performing threshold for each model. **(B)** A Venn diagram illustrating the overlap between PVP counts predicted between the PhageScanner LSTM-RNN, PhANNs, and the BLAST classifier. **(C)** A visual illustration showing the color-coded PVPs predicted by each model laid across a linear representation of the *Mycobacteriophage* PDRPxv genome.

As PhageScanner uses PHANOTATE (McNair et al., 2019) to identify ORFs, we compared the CDS locations found by PHANOTATE to those in GenBank, using a threshold of 10 base pairs for the predicted start and end locations. We then compared the F1 Scores for both the PhageScanner LSTM-RNN and PhANNs across different probability cutoff thresholds, focusing on the true positives identified by PHANOTATE. This analysis helped determine the optimal balance between recall and precision for each model (Figure 7A). This showed that the models had lower precision with no probability cutoffs and using higher probability cutoffs effectively reduced the false positive rate for the models. The highest performing probability cutoff thresholds were used in the final analysis for both the PhageScanner LSTM-RNN and PhANNs.

For the CDS regions correctly identified by PHANOTATE, most of the PVP CDS regions were correctly annotated by all three models. However, the BLAST classifier struggled to annotate non-PVP regions, leading to a significant number of false positives. This is expected as the BLAST score was not used to tune the BLAST classifier. The PhageScanner LSTM-RNN and PhANNs models accurately predicted the correct PVP classes for all coding sequences, except for two of the nine labeled with different annotations (Figure 7B). The LSTM model incorrectly identified Gp2 (Terminase Large Subunit) as a Major Capsid, while PhANNs incorrectly labeled Gp12 (Major Capsid) as a non-PVP (Figure 7C). All three models erroneously predicted Gp42 as being a minor tail protein, despite it being cataloged in GenBank as “Hypothetical.” However, an NCBI BLAST search using the Gp42 sequence revealed significant alignments to many Mycobacterium Phage proteins labeled as minor tail proteins.

##### 3.4.1.3 Testing PhageScanner on uncharacterized bacteriophage genomes

We also predicted PVPs along six uncharacterized bacteriophage genomes retrieved from the Infrastructure for a Phage Reference database (Cook et al., 2021). To do this, we used six genomes sourced from the open-access archive of 25,152 phage genomes from the Phage Reference database (Cook et al., 2021). The genome accessions we analyzed were AC171169, BK010471, GU339467, MF417929, MH552500, and MH616963. Each genome was input into the prediction pipeline as a multi-fasta file, and subsequent analyses were performed on an output prediction CSV file. Table 4 shows the counts of each of the ten PVP classes among each genome.

**Table 4:** Predicted phage virion protein (PVP) counts across bacteriophage genomes.

	**AC171169**	**BK010471**	**GU339467**	**MF417929**	**MH552500**	**MH616963**
Baseplate	3	16	9	5	8	13
Collar	0	0	20	4	4	1
HTJ	6	35	10	10	37	37
MajorCapsid	0	1	0	1	0	0
MajorTail	2	7	15	1	10	12
MinorCapsid	10	19	12	6	15	18
MinorTail	3	9	16	6	11	5
Portal	2	4	8	4	5	2
TailFiber	7	8	5	13	16	11
Shaft	2	7	5	3	1	6

Summary outlining the count of various PVP classes in each uncharacterized bacteriophage genome retrieved from the Phage Reference database (Cook et al., 2021).

**Figure 8:**
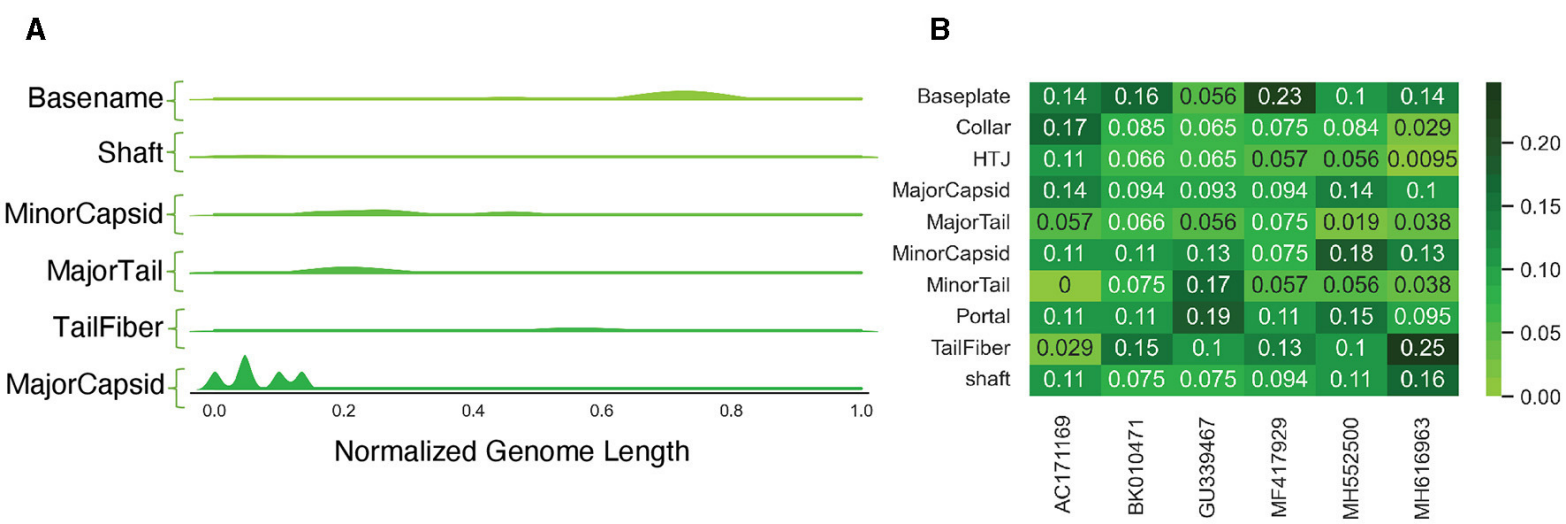
Analysis of PVPs in uncharacterized genomes. **(A)** A Kernel Density Estimation (KDE) distribution for each predicted class, aggregated along the normalized length of all genomes. **(B)** The frequency of different PVP classes within each genome, calculated on a per-genome basis.

Through our analysis, we found that most of the PVP classes tended to be located in specific regions across each genome, with the Major Capsid class typically appearing at the start of the genomes (Figure 8A). However, when we examined the occurrence of different PVP classes within each genome, we did not find a consistent pattern (Figure 8B), possibly due to our limited sample size. We additionally tallied each class per genome and revealed that some PVPs like tail fiber proteins were commonly observed throughout the genomes, while others like the Major Capsid protein were not observed frequently (see S1 Text for PVP counts per genome).

#### 3.4.2 Binary PVP prediction

For the binary prediction model which determines if a protein is a PVP or not, the BLAST classifier outperformed the learning methods with a mean F1 score of 94% (Figure 4B-top). Each PVP binary classification model was incorporated as an option in PhageScanner by referencing model descriptions in the corresponding manuscripts. Both re-implementations of PVP-SVM and LSTM showed comparable performance with an average F1 score of 91%. Furthermore, all models surpassed the baseline classifier that uses logistic regression. As seen with the multi-class prediction models, the BLAST classifier took the longest for inference, while all other classifiers ran in less than a second on the test datasets (Figure 4B-bottom).

#### 3.4.3 Finding phage toxins

Phages can be used as an effective alternative to antibiotics for treating bacterial infections, but there are safety concerns that need to be assessed. For one, there have been instances where phages aid in the pathogenicity of bacteria. Examples of this phenomenon occurring in nature include *Streptococcus pyogenes* and *Vibrio cholerae* developing toxigenicity after the induction of toxin-encoding phages (Broudy and Fischetti, 2003; Verheust et al., 2010). Here, we use PhageScanner to develop models that can predict the presence of toxic proteins from phage genomes.

**Figure 9:**
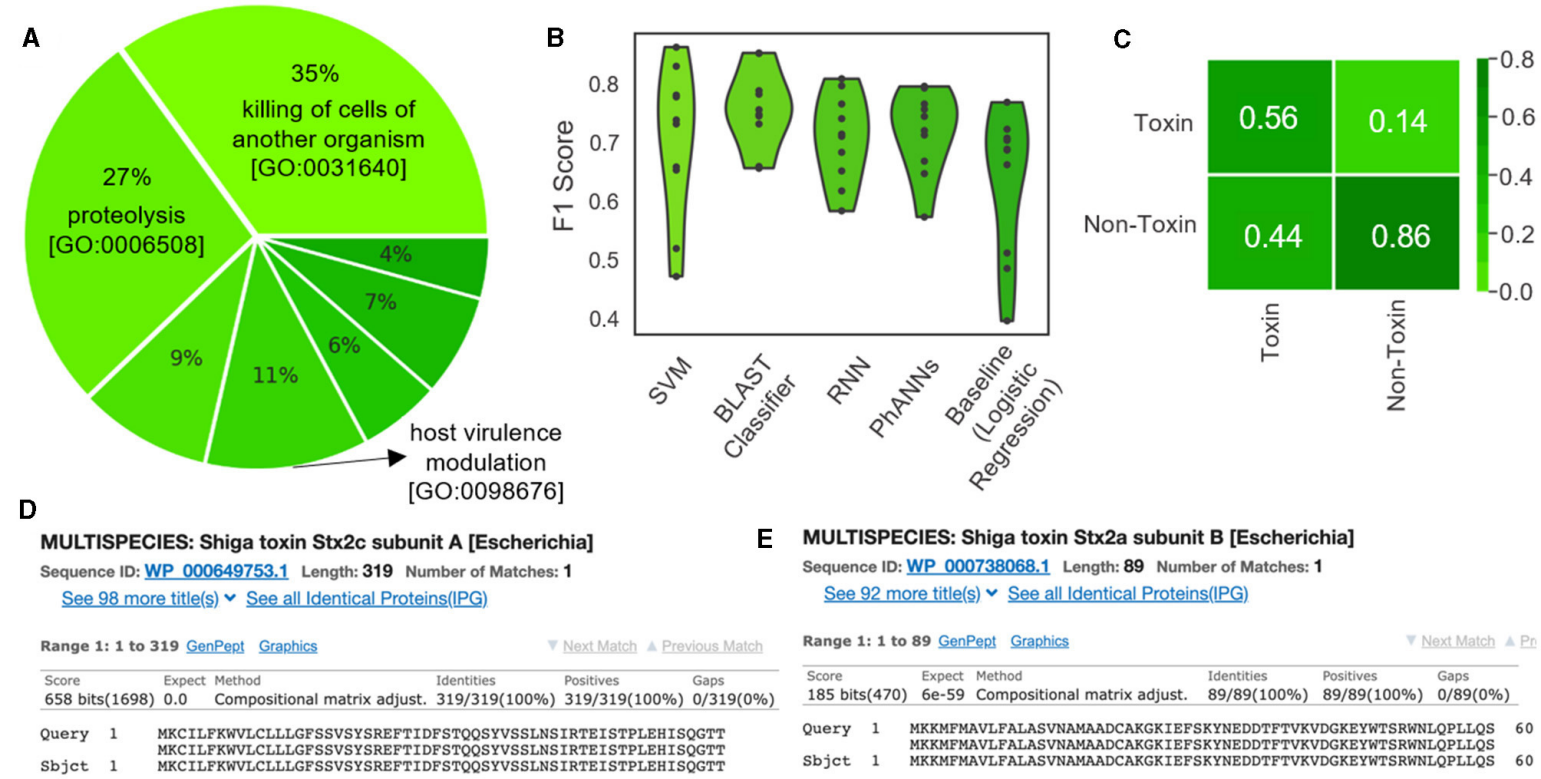
Using PhageScanner to create models for predicting phage-encoded toxins within phage genomes. **(A)** A pie chart depicting the distribution of the biological functions and gene ontologies within the training data set for the positive class. The top 3 functions and their gene ontology (GO) IDs are “killing of cells of another organism [GO: 0031640]”(35%), “proteolysis [GO:0006508]”(27%), and “modulation of host virulence by virus [GO:0098676]”(11%). The remaining functions (not labeled on the chart) are, respectively: “hemolysis by symbiont of host erythrocytes [GO: 0019836]” (9%), “defense response to bacterium [GO:0042742]” (7%), “defense response to Gram-positive bacterium [GO:0050830]” (6%), and “arachidonic acid secretion [GO:0050482]” (4%). **(B)** F1 Scores from several prediction models tasked with determining if a protein is a phage-encoded toxin. These scores come from the PhageScanner BLAST classifier, a support vector machine (SVM), a long short-term memory (LSTM) network, and a feed-forward neural network (FFNN). **(C)** Confusion matrix for the BLAST classifier comparing true labels (rows) and predicted labels (columns). **(D)** BLAST web server screenshot validating the Stx1 (Shiga toxin 1) translated protein. **(E)** BLAST web server screenshot validating the Stx2 (Shiga toxin 2) translated protein.

Verheust et al. (2010) curated a set of phage-encoded toxins associated with human diseases. We used this set, shown in Table 5, to train ML models to predict toxins within phage genomes. Specifically, nine phage-encoded toxins were defined in this set, and we established Uniprot queries for downloading each type. We also defined a negative class (i.e., non-toxin), consisting of all reviewed phage proteins that are not associated with toxicity according to the gene ontology database (Consortium, 2004) (Figure 9A; see https://github.com/Dreycey/PhageScanner for the query files). Following this process, we collated a set of 857 toxin proteins for the positive class (i.e., Toxin), and 1989 non-toxin proteins for the negative class (i.e., non-Toxin).

**Table 5:** Bacteriophage-encoded toxins.

**Phage toxin**	**Disease**	**Uniprot count**	**Uniprot query**
CholeraToxin	Cholera	14	“Cholera toxin” AND CTX
ExotoxinCspeC	Scarlet fever	71	“exotoxin C” AND (go:0090729)
ExotoxinA-speA	Scarlet fever	4	“exotoxin A” AND “(SpeA)” AND (go:0090729)
Verotoxin	Hemorrhagic diarrhea	192	“Verotoxin” OR “shiga-like toxin” AND (go:0090729)
BotulinumToxin	Botulism	11	“Botulinum toxin” AND (go:0090729)
DiptheriaToxin	Diphtheria	27	“Diphtheria toxin” AND (go:0090729)
ToxicShockProtein	Toxic shock syndrome	16	“Toxic shock” AND (go:0090729)
Cytotoxin-Ctx	Nosocomial infections and sepsis	59	“Ctx” AND (go:0090729)
ShigellaToxin	Shigellosis	2	“Shiga toxin Stx” AND (go:0090729)
GeneralToxin	NA	33	bacteriophage AND (go:0090729)
Non-Toxin	NA	401	bacteriophage NOT (go:0090729) AND reviewed: true

Overview of the bacteriophage-encoded toxins used for curating the toxin-positive training and testing set. Each toxin is presented, along with the associated diseases and the Uniprot protein count and query for each.

We tested the PhageScanner's BLAST classifier, a support vector machine (SVM), a long short-term memory (LSTM) network, and a feed-forward neural network (FFNN), using logistic regression as a baseline model for comparison. As a positive control for these models, we tested PhageScanner's BLAST classifier and FFNN on coliphage 933W (NCBI ID: NC_000924.1) which includes Shiga toxins 1 and 2 (Stx1 and Stx2). 933W contributes to the pathogenic potential of *Escherichia coli* O157:H7 (Plunkett et al., 1999; Del Cogliano et al., 2018).

In Figure 9B, we present a violin plot of the F1 scores for the four provided PhageScanner models and PhANNs (FFNN). All models displayed lower F1 scores compared to the multiclass and binary PVP prediction models (80% compared to 94%) (Figure 9B). The BLAST classifier and PhANNs had the highest average F1 scores, indicated by the widened area of the violin plot (Figure 9B).Furthermore, false positives were observed more frequently than false negatives by the BLAST classifier. This is desirable as false negatives would cause potentially toxic proteins to be missed (Figure 9C).

Then, the GUI allows for the final prediction CSV to be visually scanned for toxin-focused feature annotations. Once completed, this reveals the Stx1 and Stx2 ORFs as predicted known toxins; this is true for both the FFNN classifier and the BLAST classifier. To confirm these predictions, the Stx1 and Stx2 proteins in the expected region of the toxins were validated using the BLAST web server (Ye et al., 2006) (Figures 9D, E).

### 3.5 Summary of experimental results

The following section summarizes the results for each experiment.

- Binary and Multiclass PVP prediction: The BLAST classifier surpasses all other PVP prediction methods in terms of accuracy (94%). However, this is at the cost of efficiency as it has the highest inference time (between 20–30 seconds, dependent on datasize). See Figure 4.
- Characterized Bacteriophage Genomes: The BLAST classifier surpasses the LSTM model with these characterized proteins (Figure 4). The LSTM model struggles with misclassifying certain classes such as incorrectly predicting Portal proteins as Minor Capsid proteins (Figure 6A). These particular misclassifications occur in other models such as PhANNs (Cantu et al., 2020).
- Mycobacteriophage PDRPx Genome: Provided higher probability cutoff thresholds, the models (LSTM-RNN, PhANNs, and BLAST Classifier) had lower false positive rates. The BLAST Classifier struggled with the non-PVP regions, while the LSTM-RNN and PhANNs models were more successful in predicting the correct PVP classes for all PHANOTATE-identified coding sequences. See Figure 7.
- Uncharacterized Bacteriophage Genomes: Possibly due to small sample size, there were no consistent patterns regarding the appearance of different PVP classes within certain regions. However, there were some PVPs that were more commonly observed in all genomes. See Figure 8.
- Phage Toxin Prediction: Several models were tested with a positive control for phage toxin prediction, but their overall performance (measured by F1 score) was worse than the previous task (PVP prediction) (Figure 9B). The confusion matrix shows that the BLAST classifier has some confusions (notably, it classifies non-toxins as toxins 44% of the time) (Figure 9C). This implies there needs to be more work done (either through training on additional data and/or hyperparameter tuning) to improve the reliability of these models.

## 4 Discussion

The concerns around phage therapy include aspects such as phage selection, phage host-range limitations, and unfamiliarity with phages (Loc-Carrillo and Abedon, 2011). Phage selection, or the ability to select phages with high potential for killing the target bacteria and low potential to harm the host organism, is of critical importance to the acceptance and success of phage therapy as a viable treatment. PhageScanner's ability to predict PVPs in characterized and uncharacterized genomes are crucial to understanding how phages interact with their bacterial host and thus, can assist in better informed decisions around selecting the right phage for therapeutic use. Also, scientists' ability to predict phage-encoded toxins can guide the creation of safety protocols for future applications of phage therapy.

PhageScanner introduces a framework that provides a strong foundation for developing new protein annotation models. Specifically, we developed the framework to allow different types of protein feature annotations to occur simultaneously. Moreover, PhageScanner supports many models and thus enables cross-referenced predictions. For example, models can be used to predict if an ORF consists of a PVP protein and/or a toxin. In doing so, we present the first work which applies these PVP prediction models as a foundation for phage genome annotation. As a proof-of-concept illustrating this feature, we developed models for predicting phage-encoded proteins with bacteriophage genomes (or metagenomic data; not shown) as input.

PhageScanner is structured to be modular and address data curating, model training and PVP prediction as schematically depicted in Figure 1. When running on its default mode, this framework allows users to assign proteins to predefined classes and curate each class from their choice of the integrated databases (UniProt, Entrez, or both) prior to training their chosen models. Apart from the default mode, the user has the opportunity to reconfigure PhageScanner at model level, by providing a specific list of features, or even integrating their own model. We intentionally designed PhageScanner to facilitate comparisons between ML-based methods and our in-house BLAST-based classifier. The rationale behind this is to promote community engagement, with the projection that the more accessible the research tools are for lab technicians to use in their experimental design, the faster we can advance the computational methods for biology. We directly outline the capabilities of PhageScanner to previously mentioned tools in Table 6.

**Table 6:** Comparison table of PhageScanner capabilities.

**Tool name**	**Classification modes**		**Input types accepted**		
	**Binary PVP detection**	**Multiclass PVP detection**	**Full genome annotation**	**Metagenomic sequencing**	**Proteins**
PhageScanner^*^	X	X	X	X	X
PhANNs^*^		X			X
DeePVP	X	X			X
PVP-SVM	X				X
SCORPION	X				X
Naive Bayes Classifier	X				X
iVIRIONs	X				X

Classification modes and input types accepted for PhageScanner and other well-known tools for PVP prediction. Tools with an asterisk “*” denote that the tool has an available Github with source code, enabling the user to run the tool locally and that the models can be retrained on a new dataset.

Going forward, our plan is to focus on enhancing the performance of PhageScanner with the goal of making it highly efficient for use on high-performance computing systems. Further improvements that can be implemented include providing more refined and optimized models (for PVP and phage toxin prediction) for users to characterize samples with multiple bacteriophage genomes, inclusion of the PHROGs database (Terzian et al., 2021), to enhance the set of proteins used for PVP prediction, and creating a web-based application to allow users to upload their samples to a community platform and receive annotations.

As mentioned, PhageScanner is open-source and can be freely accessed and used, and it is licensed under the GNU General Public License. The project is hosted on GitHub at the following link: https://github.com/Dreycey/PhageScanner. The GitHub page also offers options for subscribing or unsubscribing to the PhageScanner mailing list. Since PhageScanner is an open-source software, we anticipate that community feedback will play a crucial role in steering PhageScanner's future development.

## Data Availability

The original contributions presented in the study are included in the article/supplementary material, further inquiries can be directed to the corresponding author.
